# Prognostic Significance and Primary–Metastatic Differences in VASH1 Expression, CD34-Defined Microvessel Density, and VEGF Expression in Colorectal Cancer

**DOI:** 10.3390/ijms27146319

**Published:** 2026-07-16

**Authors:** Oktay Halit Aktepe, Rezan Berkay Izgor, Ozlem Aydin Isak, Olcay Kurtulan, Tugce Ulasli, Suayib Yalcin

**Affiliations:** 1 Department of Medical Oncology, Dokuz Eylul University, Izmir 35330, Türkiye; 2 Department of Internal Medicine, Faculty of Medicine, Dokuz Eylul University, Izmir 35330, Türkiye; 3 Department of Medical Oncology, Ankara Etlik City Hospital, Ankara 06170, Türkiye; 4 Department of Pathology, Faculty of Medicine, Hacettepe University, Ankara 06230, Türkiye; 5 Department of Medical Oncology, Hacettepe University Cancer Institute, Ankara 06230, Türkiye

**Keywords:** angiogenesis, colorectal cancer, microvessel density, prognostic biomarkers, vasohibin-1

## Abstract

This study evaluated the prognostic significance of vasohibin-1 (VASH1) expression, cluster of differentiation 34 (CD34)-defined microvessel density (MVD), and vascular endothelial growth factor (VEGF) expression, as well as the differences in these parameters between primary and metastatic colorectal cancer (CRC) lesions. Tissue microarrays were used to quantify VASH1 and VEGF expression and CD34-defined MVD. Receiver operating characteristic (ROC) analysis identified optimal cut-off values for overall survival (OS). Correlations among markers were analyzed with Spearman’s test, paired tissue comparisons by the Wilcoxon signed-rank test, and survival outcomes by Kaplan–Meier and Cox regression analyses. The study included 144 CRC patients (median age: 60 years; 59% male). ROC analysis determined optimal thresholds of 6 for VASH1 (area under the curve [AUC]: 0.79, 95% confidence interval [CI]: 0.72–0.87), 37 for CD34-defined MVD (AUC: 0.76, 95% CI: 0.68–0.84), and 6 for VEGF (AUC: 0.67, 95% CI: 0.58–0.76). Patients with high VASH1 expression, high CD34-defined MVD, and high VEGF expression had significantly shorter OS compared with their corresponding low-marker groups (VASH1, *p* < 0.001; CD34-defined MVD, *p* < 0.001; VEGF, *p* = 0.007). Among the 45 patients with paired primary and metastatic samples, metastatic lesions showed significantly lower VASH1 and VEGF expression and lower CD34-defined MVD than matched primary tumors, with median values decreasing from 6.0 to 4.0 for VASH1 (*p* = 0.002), from 4 to 3 for VEGF (*p* < 0.001), and from 32 to 27 for CD34-defined MVD (*p* < 0.001). In multivariate analysis, high VASH1 expression (hazard ratio [HR]: 2.05, 95% CI: 1.01–4.20, *p* = 0.048) and high CD34-defined MVD (HR: 2.12, 95% CI: 1.15–3.89, *p* = 0.015) remained independent predictors of poor OS. High VASH1 expression and high CD34-defined MVD were independent adverse prognostic factors for OS in CRC. Lower angiogenesis-related marker levels in metastatic lesions suggest heterogeneity in tumor angiogenesis. External validation is required before clinical application.

## 1. Introduction

Colorectal cancer (CRC) is one of the most frequent malignancies and a leading cause of cancer-related mortality worldwide [1]. Despite advances in surgery and systemic therapy, survival outcomes in advanced disease remain unsatisfactory, mainly due to tumor heterogeneity, invasion, and metastatic potential [2,3].

Tumor angiogenesis contributes to CRC progression by providing the vascular support required for tumor proliferation and metastatic spread. Microvessel density (MVD), commonly assessed by immunohistochemical (IHC) staining of endothelial markers such as cluster of differentiation 34 (CD34), cluster of differentiation 31 (CD31), or factor VIII-related antigen [4], serves as a morphologic marker reflecting the degree of angiogenic activity within the tumor microenvironment. Among these markers, CD34 is the most widely utilized endothelial marker due to its robust endothelial selectivity and consistent staining patterns. Several studies in CRC have reported that higher CD34-based MVD has been linked to nodal involvement, advanced stage, and poorer prognosis [5,6,7,8].

Vasohibin-1 (VASH1) is an endothelial cell-derived protein that is upregulated by vascular endothelial growth factor (VEGF) and fibroblast growth factor-2 and acts as an intrinsic inhibitor of angiogenesis [9]. While VASH1 serves as a negative regulator of angiogenesis under physiological conditions, its overexpression within tumor-associated endothelium has paradoxically been linked to enhanced tumor aggressiveness and unfavorable prognosis in several malignancies, including CRC [10,11,12,13]. The paradoxical increase in VASH1 expression may indicate its role as a feedback marker of excessive angiogenic stimulation and vascular remodeling, rather than a true anti-angiogenic effect [14]. VEGF, a key mediator of tumor angiogenesis, promotes endothelial activation, proliferation, and vascular permeability [15]. Its overexpression has been associated with poor prognosis in CRC and provides the biological rationale for anti-VEGF strategies, including bevacizumab, in the metastatic setting [16].

Given the intricate balance between pro- and anti-angiogenic mechanisms, concurrent evaluation of VASH1 expression, CD34-defined MVD, and VEGF expression may provide a more integrated understanding of angiogenic regulation and its potential prognostic implications in CRC. Thus, the present study aimed to examine VASH1 and VEGF expression and CD34-defined MVD in CRC and to determine their associations with clinicopathological characteristics and overall survival (OS). In addition, paired analyses of primary and metastatic tumor samples were performed, where available, to explore spatial differences in angiogenesis-related marker expression.

## 2. Results

### 2.1. Patient Characteristics and Marker Cut-Off Determination

A total of 144 patients who underwent colectomy for CRC were included in the study. Baseline clinicopathologic characteristics of all patients are shown in Table 1. The median age was 60 years (interquartile range [IQR], 57–65), and 59% of the patients were male. Most tumors were located on the left side of the colon (78.5%), and moderately-to-poorly differentiated histology was observed in 53.5% of cases. According to the American Joint Committee on Cancer (AJCC) stage grouping, 4 patients (2.8%) had stage I disease, 22 (15.3%) had stage II disease, 50 (34.7%) had stage III disease, and 68 (47.2%) had stage IV disease. Lymphovascular invasion (LVI) and perineural invasion (PNI) were each present in 69.4% of cases. All patients had microsatellite-stable (MSS) disease, and *KRAS* mutation was identified in 56.3% of the cohort.

**Figure 1:**
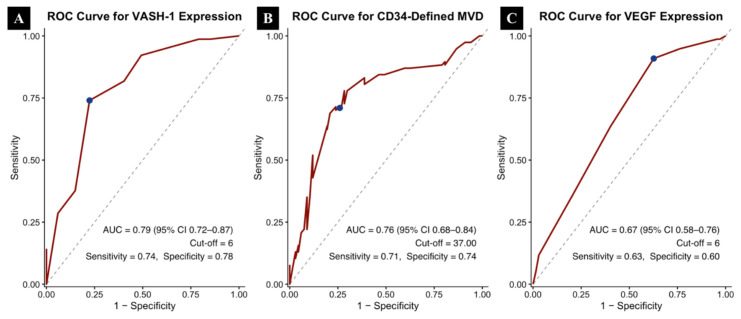
ROC curve analysis demonstrating the optimal cut-off values of VASH1 expression, CD34-defined MVD, and VEGF expression for predicting OS. Representative IHC images presented in Figure 2 and Figure 3 illustrate the scoring approach used for angiogenic markers in CRC tissue. Abbreviations: AUC: area under the curve; CD34: cluster of differentiation 34; CI: confidence interval; CRC: colorectal cancer; IHC: immunohistochemistry; MVD: microvessel density; OS: overall survival; ROC: receiver operating characteristic; VASH1: vasohibin-1; VEGF: vascular endothelial growth factor.

As shown in Figure 1, receiver operating characteristic (ROC) curve analysis identified the optimal cut-off values for predicting OS as 6 for VASH1 (area under the curve [AUC]: 0.79, 95% confidence interval [CI]: 0.72–0.87; sensitivity: 74%, specificity: 78%, Figure 1A), 37 for CD34-defined MVD (AUC: 0.76, 95% CI: 0.68–0.84; sensitivity: 71%, specificity: 74%, Figure 1B), and 6 for VEGF (AUC: 0.67, 95% CI: 0.58–0.76; sensitivity: 63%, specificity: 60%, Figure 1C). According to these cut-off values, patients were subsequently classified into low- and high-marker groups for further prognostic evaluation.

**Figure 2:**
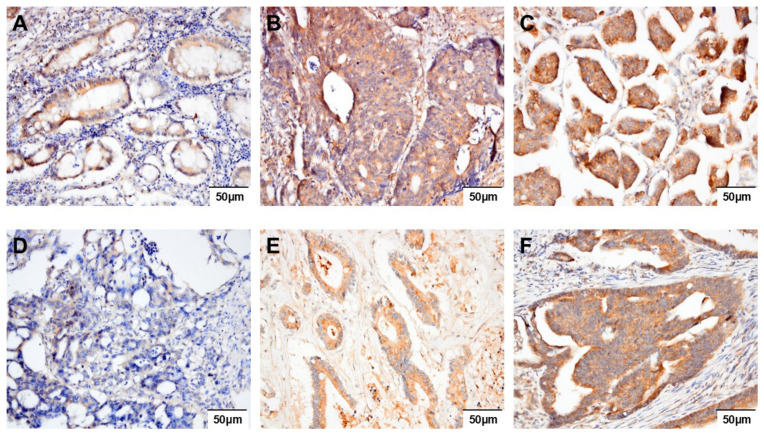
Representative IHC staining of VASH1 (**A**–**C**) and VEGF (**D**–**F**) in CRC tissues. The staining intensity for each marker was classified as weak ((**A**,**D**); 1+), moderate ((**B**,**E**); 2+), and strong ((**C**,**F**); 3+) (×400). Abbreviations: CRC: colorectal cancer; IHC: immunohistochemistry; VASH1: vasohibin-1; VEGF: vascular endothelial growth factor.

**Figure 3:**
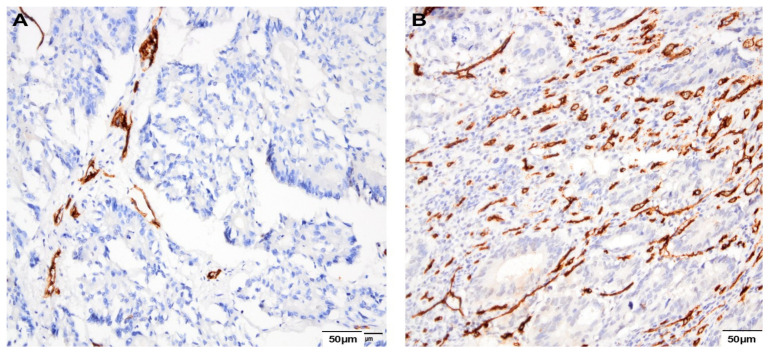
Anti-CD34 immunostaining illustrating low (**A**) and high (**B**) MVD in CRC (×200). Abbreviations: CD34: cluster of differentiation 34; CRC: colorectal cancer; MVD: microvessel density.

Figure 2 shows weak (1+), moderate (2+), and strong (3+) staining patterns for VASH1 and VEGF, whereas Figure 3 demonstrates low- and high-MVD according to CD34 immunostaining.

Half of the tumors demonstrated high VASH1 expression (n = 72, 50%). As shown in Table 1, there were no significant differences between the VASH1-high and VASH1-low groups in terms of age, sex, tumor location, *KRAS* status, or nodal involvement. However, VASH1 expression was significantly associated with the presence of distant metastasis (M1: 65.3% vs. 29.2%, *p* < 0.001) and poorer histologic differentiation (moderate-to-poor: 63.9% vs. 43.1%, *p* = 0.012). LVI was numerically more frequent in the VASH1-high group, while PNI was numerically less frequent in the VASH1-high group; however, neither association reached statistical significance (LVI, *p* = 0.148; PNI, *p* = 0.469).

**Table 1:** Baseline clinicopathologic patient characteristics stratified according to VASH1 expression status.

		VASH1 Expression		
Variable	All Patients n = 144	Low n = 72 (50%)	High n = 72 (50%)	*p* Value
Sex				0.865
male	85 (59%)	43 (59.7%)	42 (58.3%)	
female	59 (41%)	29 (40.3%)	30 (41.7%)	
Age, years, median (IQR)	60 (57–65)	60 (56–64)	61 (58–66)	0.415
T stage				0.065
T1–3	81 (56.3%)	46 (63.9%)	35 (48.6%)	
T4	63 (43.8%)	26 (36.1%)	37 (51.4%)	
Lymph node status				0.348
N0	39 (27.1%)	22 (30.6%)	17 (23.6%)	
N1–2	105 (72.9%)	50 (69.4%)	55 (76.4%)	
*KRAS* mutation status				0.614
wild	63 (43.8%)	33 (45.8%)	30 (41.7%)	
mutant	81 (56.3%)	39 (54.2%)	42 (58.3%)	
M0	76 (52.8%)	51 (70.8%)	25 (34.7%)	<0.001
M1	68 (47.2%)	21 (29.2%)	47 (65.3%)	
LVI				0.148
absent	44 (30.6%)	26 (36.1%)	18 (25%)	
present	100 (69.4%)	46 (63.9%)	54 (75%)	
PNI				0.469
absent	44 (30.6%)	20 (27.8%)	24 (33.3%)	
present	100 (69.4%)	52 (72.2%)	48 (66.7%)	
Tumor location				0.839
left	113 (78.5%)	56 (77.8%)	57 (79.2%)	
right	31 (21.5%)	16 (22.2%)	15 (20.8%)	
Histology				0.012
good	67 (46.5%)	41 (56.9%)	26 (36.1%)	
moderate-poor	77 (53.5%)	31 (43.1%)	46 (63.9%)	

Abbreviations: IQR: interquartile range; LVI: lymphovascular invasion; PNI: perineural invasion; VASH1: vasohibin-1.

As shown in Table 2, high CD34-defined MVD was observed in 75 patients (52.1%). Similarly to VASH1, CD34-defined MVD did not differ significantly by age, sex, tumor site, or *KRAS* mutation status. However, tumors with high CD34-defined MVD tended to present with more advanced disease, including a higher frequency of distant metastasis (M1: 54.7% vs. 39.1%, *p* = 0.062) and poorer histological differentiation (moderate-to-poor: 61.3% vs. 44.9%, *p* = 0.049). LVI and PNI were more common among patients with high CD34-defined MVD, although these differences were not statistically significant. However, VEGF expression showed no significant association with any of the assessed clinicopathological characteristics (all *p* > 0.05).

### 2.2. Correlation and Comparative Analysis of Angiogenic Markers

In the overall paired cohort (n = 45), metastatic lesions were located in the liver in 29 patients (64.4%), lung in 8 patients (17.8%), ovary/adnexa in 7 patients (15.6%), and brain in 1 patient (2.2%). For site-based comparisons, non-liver metastatic lesions were grouped as extrahepatic metastases. Metastatic angiogenesis-related marker levels did not differ significantly between liver and extrahepatic metastases. Median metastatic VEGF expression was 3.0 (IQR, 1.5–4.0) in liver metastases and 3.0 (IQR, 2.0–6.0) in extrahepatic metastases (*p* = 0.556). Median CD34-defined MVD was 30.0 (IQR, 19.0–39.0) and 23.0 (IQR, 18.0–28.0), respectively (*p* = 0.089), while median VASH1 expression was 4.0 (IQR, 2.0–8.0) and 3.5 (IQR, 2.0–4.0), respectively (*p* = 0.192).

As shown in Table 3, Spearman correlation analysis demonstrated significant positive correlations among the angiogenic markers in both primary and metastatic tissues. In primary tumors, VASH1 expression was positively correlated with VEGF expression (r = 0.423, *p* < 0.001) and CD34-defined MVD (r = 0.538, *p* < 0.001), while VEGF expression and CD34-defined MVD also showed a weaker but significant positive correlation (r = 0.276, *p* < 0.001). A similar pattern was observed in metastatic tissues, where VASH1 expression remained positively correlated with VEGF expression (r = 0.354, *p* = 0.017) and CD34-defined MVD (r = 0.612, *p* < 0.001), and VEGF expression was also correlated with CD34-defined MVD (r = 0.297, *p* = 0.048). Overall, these findings indicate biologically coherent and interrelated angiogenesis-related marker patterns across both primary and metastatic CRC tissues.

In the paired cohort, metastatic tissues showed lower angiogenesis-related marker levels than matched primary tumors, with median VASH1 decreasing from 6.0 (IQR, 3–8) to 4.0 (IQR, 2–6), VEGF from 4 (IQR, 3–8) to 3 (IQR, 2–5), and CD34-defined MVD from 32 (IQR, 22–51) to 27 (IQR, 18–34). As presented in Table 4, these differences were statistically significant in Wilcoxon signed-rank analyses (VASH1: Z = −3.094, *p* = 0.002; VEGF: Z = −3.652, *p* < 0.001; CD34-defined MVD: Z = −4.476, *p* < 0.001). In subgroup analyses, patients with adjuvant recurrence also demonstrated significantly lower metastatic VASH1 expression (Z = −2.542, *p* = 0.011), VEGF expression (Z = −2.832, *p* = 0.005), and CD34-defined MVD (Z = −4.126, *p* < 0.001). However, among patients with de novo metastatic disease, only VEGF expression remained significantly lower in metastatic tissue (Z = −2.382, *p* = 0.017), whereas the differences for VASH1 expression (Z = −1.782, *p* = 0.075) and CD34-defined MVD (Z = −1.874, *p* = 0.061) did not reach statistical significance.

### 2.3. Survival Outcomes

**Figure 4:**
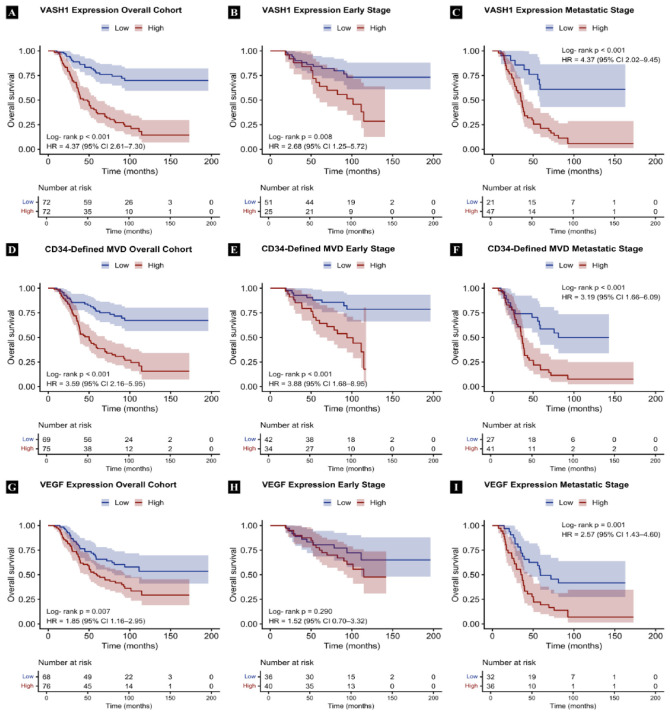
Kaplan–Meier curves for OS according to VASH1 expression, CD34-defined MVD, and VEGF expression in the overall cohort, early-stage subgroup, and metastatic subgroup. (**A**–**C**) VASH1 expression in the overall cohort, early-stage subgroup, and metastatic subgroup, respectively; (**D**–**F**) CD34-defined MVD in the overall cohort, early-stage subgroup, and metastatic subgroup, respectively; (**G**–**I**) VEGF expression in the overall cohort, early-stage subgroup, and metastatic subgroup, respectively. HRs with 95% CIs and log-rank *p* values are displayed within each panel. HRs were estimated using univariate Cox proportional hazards models for the corresponding high- versus low-marker comparison. Abbreviations: CD34: cluster of differentiation 34; CI: confidence interval; HR: hazard ratio; MVD: microvessel density; OS: overall survival; VASH1: vasohibin-1; VEGF: vascular endothelial growth factor.

The median OS for the cohort was 90 months (95% CI: 64.1–115.9). A total of 77 deaths (53.5%) were recorded during a median follow-up of 100.9 months (95% CI: 92.8–109.0). Figure 4 illustrates Kaplan–Meier survival curves stratified by VASH1 expression, CD34-defined MVD, and VEGF expression in the overall, early-stage, and metastatic-stage patient groups. Patients with high VASH1 expression had significantly poorer OS than those with low expression in the overall cohort (43.0 months, 95% CI: 30.2–55.8 vs. not reached, *p* < 0.001, Figure 4A). This pattern was maintained in both stage-based subgroups: in early-stage disease, median OS was not reached in the low-VASH1 group and was 101.4 months (95% CI: 67.5–135.1) in the high-VASH1 group (*p* = 0.008, Figure 4B), while in metastatic disease, median OS was not reached in the low-VASH1 group versus 35.1 months (95% CI: 31.7–38.4) in the high-VASH1 group (*p* < 0.001, Figure 4C). In the overall cohort, high CD34-defined MVD was associated with significantly poorer OS than low CD34-defined MVD (50.1 months, 95% CI: 37.2–62.9 vs. not reached, *p* < 0.001, Figure 4D). This pattern was maintained in both stage-based subgroups, with median OS of 93.9 months (95% CI: 62.7–125.0) in the high CD34-defined MVD group versus not reached in the low CD34-defined MVD group in early-stage disease (*p* < 0.001, Figure 4E), and 36.5 months (95% CI: 33.4–39.5) versus 81.0 months (95% CI not estimable), respectively, in the metastatic subgroup (*p* < 0.001, Figure 4F). In the overall cohort, high VEGF expression was associated with significantly poorer OS than low VEGF expression (58.8 months, 95% CI: 38.5–79.2 vs. not reached, *p* = 0.007, Figure 4G), whereas in the early-stage subgroup the difference was not statistically significant, although median OS was shorter in the high-expression group (114.5 months, 95% CI: not estimable vs. not reached, *p* = 0.290, Figure 4H). However, in the metastatic subgroup, high VEGF expression was associated with significantly poorer OS, with median OS of 35.0 months (95% CI: 24.4–45.6) versus 58.7 months (95% CI: 29.8–87.6) in the low-expression group (*p* = 0.001, Figure 4I).

As shown in Table 5, univariate Cox regression analysis identified several variables that were significantly associated with OS. Patients with distant metastasis had markedly poorer OS (hazard ratio [HR]: 3.51, 95% CI: 2.18–5.64, *p* < 0.001). Similarly, high VASH1 expression (HR: 4.37, 95% CI: 2.61–7.30, *p* < 0.001), high CD34-defined MVD (HR: 3.59, 95% CI: 2.16–5.95, *p* < 0.001), and high VEGF expression (HR: 1.85, 95% CI: 1.16–2.95, *p* = 0.009) were significantly associated with poorer OS. Histologic grade also showed prognostic relevance, with patients harboring moderate-to-poorly differentiated tumors exhibiting worse OS (HR: 1.71, 95% CI: 1.07–2.71, *p* = 0.023). As shown in Table 5, in the multivariate model, independent predictors of OS included distant metastasis (HR: 2.92, 95% CI: 1.75–4.88, *p* < 0.001), VASH1 expression (HR: 2.05, 95% CI: 1.01–4.20, *p* = 0.048), and CD34-defined MVD (HR: 2.12, 95% CI: 1.15–3.89, *p* = 0.015). VEGF expression lost statistical significance after adjustment (HR: 1.16, 95% CI: 0.66–2.03, *p* = 0.592).

As shown in Table 6, a sensitivity multivariable Cox regression analysis was performed using median-based cut-offs (VASH1: 5, VEGF: 6, and CD34-defined MVD: 38) to reduce reliance on ROC-derived thresholds. In this model, distant metastasis remained independently associated with poorer OS (HR: 2.86, 95% CI: 1.71–4.77; *p* < 0.001). Among the angiogenic markers, high VASH1 expression remained an independent predictor of worse OS (HR: 2.11, 95% CI: 1.04–4.31; *p* = 0.039), and high CD34-defined MVD showed a similar independent association (HR: 2.03, 95% CI: 1.12–3.68; *p* = 0.020). However, VEGF was not independently associated with OS in this analysis (HR: 1.17, 95% CI: 0.67–2.04; *p* = 0.592). LVI (HR: 1.23, 95% CI: 0.72–2.09; *p* = 0.448) and histology (HR: 1.18, 95% CI: 0.72–1.93; *p* = 0.512) also did not show independent prognostic significance.

## 3. Discussion

In this study, we evaluated the prognostic relevance of VASH1 expression, CD34-defined MVD, and VEGF expression in CRC and examined their patterns in matched primary and metastatic tissues. High VASH1 expression and high CD34-defined MVD were independently associated with poorer OS, supporting their role as adverse prognostic biomarkers. Although high VEGF expression was associated with shorter OS in univariate analyses, this association was not retained after adjustment. In the paired cohort, metastatic tissues showed lower VASH1 expression, VEGF expression, and CD34-defined MVD than matched primary tumors. This finding suggests angiogenesis-related heterogeneity between primary and metastatic CRC lesions, but should not be interpreted as evidence of a single biological mechanism. Rather, differences in angiogenesis-related marker levels may reflect the combined effects of tumor progression, prior treatment exposure, clonal selection, molecular divergence, and metastatic-site-specific microenvironmental adaptation.

VASH1 is an endothelial cell–derived protein that is induced by pro-angiogenic stimuli, particularly VEGF, and acts as a negative feedback inhibitor of angiogenesis [9]. It is produced by activated endothelial cells at the termination phase of angiogenic sprouting, thereby functioning as a “self-defense” mechanism against excessive neovascularization [17,18]. Paradoxically, despite its inhibitory role in vitro, elevated VASH1 expression in tumor tissues has often been associated with poor prognosis in a variety of solid malignancies [12,19,20,21], suggesting that its upregulation represents a response to intensified angiogenic activity and vascular remodeling, rather than an effective inhibition of neovascularization. In CRC, VASH1 expression has been reported predominantly in endothelial cells lining microvessels within the tumor stroma, often co-localizing with CD34-positive vasculature [17]. Multiple studies have demonstrated that high VASH1 expression correlates with increased MVD, higher VEGF expression, deeper tumor invasion, nodal and distant metastasis, and unfavorable survival outcomes [22,23]. These findings indicate that VASH1 may serve as a surrogate marker of angiogenic activation and tumor aggressiveness.

MVD, determined by counting vascular hot spots through IHC staining with CD34, CD31, or Factor VIII–related antigen, is a widely used quantitative marker of intratumoral angiogenesis and indicates the extent of tumor neovascularization [4]. Notably, newly formed CD34-positive vessels are generally immature and structurally irregular, exhibiting increased permeability that facilitates tumor cell invasion and metastatic niche development [24]. Moreover, elevated angiogenic signaling is frequently accompanied by upregulation of hypoxia-inducible factor 1-alpha and VEGF, further amplifying proangiogenic cascades and resistance to apoptosis [25]. In the present study, CD34 was selected as the principal marker for MVD assessment, as it is a widely used pan-endothelial marker with reliable staining performance in formalin-fixed paraffin-embedded (FFPE) CRC tissues and allows comparison with previous CRC angiogenesis studies [4]. Although CD31 is also commonly used for endothelial identification [26], a meta-analysis of CRC studies demonstrated that MVD assessed using CD31 or CD34 was significantly associated with survival, whereas no such association was found for factor VIII, thereby supporting the prognostic reliability of CD34 in this context [4]. In contrast, endoglin preferentially marks activated endothelial cells involved in tumor angiogenesis, unlike panendothelial markers such as CD31 and CD34, which stain all vessels regardless of activation status, and therefore reflects active neoangiogenesis rather than total MVD [27].

From a therapeutic perspective, our findings should not be interpreted as indicating that VASH1 or CD34 are direct treatment targets in CRC. VASH1 is an endogenous negative feedback regulator of angiogenesis [9], whereas CD34-defined MVD reflects the extent of tumor-associated microvasculature rather than a direct oncogenic driver [4,28]. Therefore, high VASH1 expression or high CD34-defined MVD may be more appropriately considered a marker of an angiogenesis-associated tumor microenvironment. In such tumors, VEGF-pathway inhibition remains the most biologically plausible anti-angiogenic strategy, as VEGF is the established therapeutic target in metastatic CRC and bevacizumab has demonstrated survival benefit when combined with fluorouracil-based chemotherapy [29]. However, whether VASH1-high or CD34-defined MVD-high tumors derive greater benefit from anti-VEGF-based therapy remains uncertain. In the present cohort, treatment exposure was heterogeneous and the number of patients within specific treatment subgroups was limited; therefore, we could not reliably evaluate treatment-specific survival outcomes in patients with high VASH1 expression or high CD34-defined MVD. Future treatment-stratified studies are needed to determine whether these markers have predictive, rather than purely prognostic, value for anti-angiogenic therapy.

This study’s primary strength is the comprehensive evaluation of VASH1 expression, CD34-defined MVD, and VEGF expression within a uniform patient cohort, offering a deeper and more integrated perspective on tumor vascular dynamics. A follow-up period of over eight years strengthened the study by allowing a comprehensive analysis of survival outcomes. To our knowledge, this is the first study to evaluate VASH1 expression, CD34-defined MVD, and VEGF expression concurrently in paired primary and post-treatment metastatic CRC tissues. Unlike previous studies that examined only primary tumors, our analysis provides a longitudinal perspective on angiogenic changes throughout disease progression. For instance, Yan et al. investigated VASH1, CD34-defined MVD, and vascular endothelial growth factor A (VEGF-A) expression in 132 primary CRC samples and reported significant correlations among these angiogenic markers; however, only VASH1 remained independently associated with poorer OS [23]. Consistently, Liu et al. demonstrated that VASH1 exerts tumor-suppressive effects in CRC models; however, high endothelial VASH1 expression in clinical specimens was associated with enhanced angiogenesis and poorer survival [13]. Nevertheless, these earlier reports were limited to primary tumors and did not investigate angiogenic alterations in metastatic tissue or after therapeutic intervention.

Several studies have explored CD34-defined MVD or VEGF expression in CRC metastases; however, these analyses were either confined to synchronous lesions or lacked assessment of all three angiogenic markers. Jeong et al. compared VEGF-A expression and CD34-defined MVD in matched primary CRC and liver metastases, reporting higher VEGF expression and MVD in primary tumors [30]. Yin et al. found that MVD and VEGF were lower in lymph node metastases than in primaries [28]. Similarly, Eefsen et al. demonstrated that bevacizumab-treated liver metastases exhibited significantly reduced MVD compared with untreated counterparts, suggesting vascular suppression following anti-angiogenic therapy [31]. Moreover, Okamoto et al. examined 48 resected liver metastases of CRC after systemic chemotherapy and found that CD34-defined MVD correlated with contrast enhancement patterns on computed tomography, linking histologic vascularity with radiologic perfusion characteristics [32]. Yet, none of these studies used matched pre- and post-treatment samples, nor did they evaluated VASH1. By evaluating all three markers in a matched, post-therapy setting, our study indicates that CRC angiogenesis may evolve after systemic treatment, which may have implications for future anti-angiogenic approaches.

An alternative explanation for the reduced VASH1 expression, CD34-defined MVD, and VEGF expression observed in metastatic lesions is molecular evolution and spatial selection during CRC progression. Primary and metastatic CRC lesions may differ not only in vascular architecture but also in clonal composition, mutational profiles, stromal content, immune contexture, and organ-specific microenvironmental pressures. Oliveira et al. demonstrated that CRCs arising in different colon segments may harbor distinct mutational profiles, supporting the concept of spatial molecular heterogeneity in CRC [33]. In this context, the lower angiogenesis-related marker levels observed in metastatic lesions should not be attributed solely to prior treatment exposure or angiogenic remodeling. Instead, these differences may also reflect selection of metastatic subclones with distinct vascular and molecular phenotypes, adaptation to the metastatic organ microenvironment, or broader molecular subtype-related biology. Therefore, our paired primary–metastatic findings should be interpreted as hypothesis-generating evidence of angiogenesis-related heterogeneity, potentially shaped by clonal selection, molecular divergence, treatment exposure, and metastatic-site-specific microenvironmental adaptation, rather than as definitive proof of treatment-induced or progression-related vascular evolution.

CRC is characterized by substantial molecular heterogeneity, with recurrent alterations involving WNT/APC, RAS/RAF, phosphoinositide 3-kinase (PI3K), transforming growth factor beta (TGF-β), and p53 signaling pathways [34]. Beyond these individual gene-level alterations, transcriptome-based consensus molecular subtype (CMS) classification has identified four biologically distinct CRC subtypes, with CMS4 in particular characterized by mesenchymal differentiation, TGF-β activation, stromal invasion, and angiogenesis [35]. Therefore, angiogenesis-related marker expression may not reflect a purely vascular phenomenon, but may also represent broader molecular and tumor microenvironmental programs. In the present study, all patients had MSS disease, and *KRAS* mutation status was available for the entire cohort; thus, microsatellite instability-high/deficient mismatch repair-related heterogeneity was not a confounding factor. However, data on *BRAF* mutations, *TP53* and *APC* alterations, CMS classification, and broader tumor mutational heterogeneity were not available. Accordingly, we could not determine whether differences in VASH1 expression, CD34-defined MVD, and VEGF expression were independent of these molecular features or partly related to unmeasured molecular subtypes, particularly CMS4-like mesenchymal/angiogenic biology. These findings should therefore be interpreted as exploratory and require validation in comprehensively molecularly annotated MSS CRC cohorts.

This study has several limitations that should be acknowledged. First, its retrospective and single-center design inevitably limits causal inference and may introduce selection bias. Second, an important limitation of this study is the heterogeneity of treatment exposure before metastatic tissue sampling. Patients with recurrent disease had previously received adjuvant therapy, whereas another subgroup had already been exposed to systemic treatment for advanced disease before metastasectomy. Therefore, angiogenesis-related marker levels in metastatic lesions may have been influenced by prior treatment history. Although we were able to stratify the paired cohort by broader treatment setting (adjuvant recurrence vs. de novo metastatic disease), more granular subgroup analyses according to specific antiangiogenic or anti-epidermal growth factor receptor (EGFR) exposure, as well as multivariable models incorporating treatment-related variables, could not be performed due to the limited sample size of the metastatic cohort. This should be addressed in larger, prospectively designed studies. In addition, because paired molecular profiling of primary and metastatic lesions was not available, we could not distinguish whether the observed changes in angiogenesis-related marker levels were driven by treatment-related effects, angiogenic remodeling, clonal selection, molecular evolution, or metastatic-site-specific microenvironmental influences. Third, the use of tissue microarrays (TMAs) allowed standardized and efficient evaluation but confined the analysis to selected tumor regions, potentially underrepresenting intratumoral heterogeneity. Nevertheless, previous validation studies using the same methodology have demonstrated good concordance between TMA- and whole-section–based evaluations [36,37]. Fourth, functional or mechanistic analyses were not performed to clarify whether the associations between angiogenesis-related marker levels and prognosis reflect direct biological mechanisms or secondary effects of tumor aggressiveness. Fifth, an additional limitation is that pathologists were not blinded to tissue origin (primary vs. metastatic) during IHC scoring, as this information was required for sample identification; although evaluators were blinded to clinical and survival outcomes, awareness of tissue origin cannot be fully excluded as a potential source of assessment bias. Furthermore, although IHC scoring was performed independently by two pathologists, with discordant cases resolved by consensus, individual pre-consensus scores were not retained separately, precluding the calculation of a formal interobserver agreement statistic (kappa coefficient). Both issues should be addressed in future prospective studies. Sixth, another limitation is the use of ROC-derived cut-off values generated within the same cohort, which may introduce overfitting and overestimate prognostic performance. These thresholds should therefore be considered exploratory. Sensitivity analysis using median-based cut-offs confirmed that VASH1 expression and CD34-defined MVD remained independently associated with OS. External validation is still required before clinical application. Finally, the study population consisted exclusively of patients treated in a tertiary oncology center, which may limit the generalizability of the results.

## 4. Materials and Methods

### 4.1. Participants and Study Design

We conducted a retrospective, single-center cohort study involving patients with histologically confirmed CRC who underwent colectomy at the Department of Medical Oncology, Hacettepe University Cancer Institute (Ankara, Turkey), between January 2009 and December 2019. Surgical resections were performed either with curative intent in localized disease or for palliative purposes in metastatic cases at initial diagnosis. Patients were eligible for inclusion if they were ≥18 years of age and had adequate FFPE tumor material for IHC assessment, as well as complete clinical and follow-up data in the institutional database. Patients were excluded if they had a concurrent or prior malignancy other than non-melanoma skin cancer, received neoadjuvant therapy, or had inadequate or missing tumor material for TMA construction.

Primary tumor specimens were obtained at the time of curative-intent resection or palliative colectomy. Metastatic tissue specimens were obtained at the time of metastasectomy. Patients undergoing metastasectomy comprised two groups: (1) patients initially treated for localized disease who received adjuvant therapy, subsequently developed recurrent/metastatic disease, and underwent metastasectomy before initiation of first-line systemic therapy for metastatic disease; and (2) patients who underwent palliative colectomy at presentation, subsequently received systemic therapy, and later underwent metastasectomy. Accordingly, prior treatment exposure before metastatic tissue acquisition was heterogeneous across the cohort.

Demographic, clinical, and pathological variables were obtained from electronic medical records and pathology reports, including age at diagnosis, sex, depth of invasion (T stage), lymph node status (N stage), presence of distant metastasis (M stage), primary tumor location (right- or left-sided colon), PNI, LVI, histologic differentiation (well, moderate, poor), and *KRAS* mutation status. Tumor staging was determined according to the 8th edition of the AJCC Tumor–Node–Metastasis (TNM) classification.

Patients with metastatic disease at presentation received first-line systemic chemotherapy, including FOLFOX (5-fluorouracil/leucovorin/oxaliplatin), FOLFIRI (5-fluorouracil/leucovorin/irinotecan), or CAPOX (capecitabine/oxaliplatin), combined with targeted biologic agents according to molecular characteristics—bevacizumab for VEGF inhibition and cetuximab or panitumumab for EGFR blockade in *RAS* wild-type tumors. In addition, primary tumor sidedness (right- vs. left-sided colon) was considered in the analysis, given its known impact on first-line treatment selection and clinical outcomes in metastatic CRC.

### 4.2. Immunohistochemical Analyses

Hematoxylin–eosin (H&E)-stained slides and FFPE tissue blocks were retrieved from the archives of the Department of Pathology, Hacettepe University. After reviewing H&E slides, representative tumor areas were identified, and TMAs were constructed using 3 mm cores obtained from the corresponding FFPE blocks. TMA blocks were sectioned at 4 μm thickness. The sections were deparaffinized in Bond Dewax solution (Leica Microsystems, Wetzlar, Germany) at 72 °C and subsequently stained with anti-VASH1 rabbit polyclonal antibody (1:100; Novus Biologicals, Abingdon, UK), anti-CD34 antibody (1:150; Leica Biosystems, Deer Park, IL, USA; clone QBEnd/10), and anti-VEGF antibody (1:100; Biocare Medical, Pacheco, CA, USA; clone EP1176Y) using a Leica Bond Autostainer according to the manufacturer’s instructions. For a subset of 20 tumors, whole-slide IHC staining for VASH1, CD34, and VEGF was also performed and compared with the corresponding TMA cores to assess staining concordance.

All IHC evaluations were independently conducted by two pathologists blinded to clinical and survival data. In case of discrepancy, slides were jointly reviewed, and a consensus interpretation was reached. For anti-VASH1 and anti-VEGF evaluation, both the percentage of positive tumor cells and staining intensity were assessed semiquantitatively. The percentage of positive cells was scored as follows: 1 = 1–25%, 2 = 26–50%, 3 = 51–75%, and 4 = >75%. Staining intensity was graded as: 0 = negative, 1+ = weak, 2+ = moderate, and 3+ = strong. A staining index was then calculated by multiplying the percentage and intensity scores, yielding a total score between 0 and 12. For anti-CD34 staining, MVD was evaluated by selecting areas with the highest vascular density at low magnification. Within each selected field, individual microvessels were counted under a high-power field (×200; 20× objective and 10× ocular). Any brown-stained endothelial cell or cluster clearly separated from adjacent structures was counted as a single microvessel.

### 4.3. Statistical Analyses

Categorical and continuous variables were expressed as percentages and medians with IQRs, respectively. Differences between categorical variables were analyzed using the Chi-square or Fisher’s exact test, and continuous variables using the Mann–Whitney U test. ROC curve analysis was performed to determine the optimal cut-off values for continuous variables including VASH1 expression, CD34-defined MVD, and VEGF expression. Because ROC-derived thresholds were generated within the same cohort used for survival analysis, these cut-off values were considered exploratory. To reduce reliance on internally derived thresholds and assess the robustness of the findings, an additional sensitivity multivariable Cox regression analysis was performed using median-based cut-off values for VASH1 expression, CD34-defined MVD, and VEGF expression. Correlations among VASH1 expression, CD34-defined MVD, and VEGF expression were assessed using Spearman’s rank correlation coefficient. The Wilcoxon signed-rank test was used to compare paired angiogenesis-related marker levels between primary and metastatic lesions. OS was defined as the time from diagnosis to death from any cause. Survival probabilities were estimated using the Kaplan–Meier method, and comparisons between groups were evaluated with the log-rank test. For subgroup survival analyses, patients were categorized as early-stage disease (stage I–III) and metastatic disease (stage IV). Separate survival analyses for individual stage I, II, and III subgroups were not performed because of the limited sample size of individual non-metastatic stage categories, particularly the very small number of stage I patients. Variables with a *p* ≤ 0.20 in univariate analysis were entered into a multivariable Cox proportional-hazards regression model to identify independent prognostic factors. All statistical analyses were performed using the Statistical Package for the Social Sciences (SPSS), version 27.0 (IBM Corp., Armonk, NY, USA), and Kaplan–Meier survival curves were generated using R software version 4.6.0 (R Foundation for Statistical Computing, Vienna, Austria). Two-sided *p* values < 0.05 were considered statistically significant.

## 5. Conclusions

High VASH1 expression and high CD34-defined MVD were associated with poorer OS in this cohort of CRC patients. However, because the prognostic thresholds were derived within the same dataset and metastatic tissue sampling occurred in clinically heterogeneous treatment contexts, these findings should be considered exploratory and hypothesis-generating. External validation is needed before clinical application.

## Figures and Tables

**Table 2:** Baseline clinicopathologic patient characteristics stratified according to CD34-defined MVD status.

CD34-Defined MVD			
Variable	Low n = 69 (47.9%)	High n = 75 (52.1%)	*p* Value
Sex			0.441
male	43 (62.3%)	42 (56%)	
female	26 (37.7%)	33 (44%)	
Age, years, median (IQR)	62 (56–65)	60 (57–65)	0.664
T stage			0.950
T1–3	39 (56.5%)	42 (56%)	
T4	30 (43.5%)	33 (44%)	
Lymph node status			0.105
N0	23 (33.3%)	16 (21.3%)	
N1–2	46 (66.7%)	59 (78.7%)	
*KRAS* mutation status			0.785
wild	31 (44.9%)	32 (42.7%)	
mutant	38 (55.1%)	43 (57.3%)	
M0	42 (60.9%)	34 (45.3%)	0.062
M1	27 (39.1%)	41 (54.7%)	
LVI			0.156
absent	25 (36.2%)	19 (25.3%)	
present	44 (63.8%)	56 (74.7%)	
PNI			0.740
absent	22 (31.9%)	22 (29.3%)	
present	47 (68.1%)	53 (70.7%)	
Tumor location			0.452
left	56 (81.2%)	57 (76%)	
right	13 (18.8%)	18 (24%)	
Histology			0.049
good	38 (55.1%)	29 (38.7%)	
moderate-poor	31 (44.9%)	46 (61.3%)	

Abbreviations: CD34: cluster of differentiation 34; IQR: interquartile range; LVI: lymphovascular invasion; MVD: microvessel density; PNI: perineural invasion.

**Table 3:** Spearman correlation analysis of VASH1 expression, VEGF expression, and CD34-defined MVD in primary tumors and metastatic tissues.

Marker Pair	Primary Tumors		Metastatic Tissues	
	r	*p*	r	*p*
VASH1 vs. VEGF	0.423	<0.001	0.354	0.017
VASH1 vs. CD34-defined MVD	0.538	<0.001	0.612	<0.001
VEGF vs. CD34-defined MVD	0.276	<0.001	0.297	0.048

Abbreviations: CD34: cluster of differentiation 34; MVD: microvessel density; VASH1: vasohibin-1; VEGF: vascular endothelial growth factor.

**Table 4:** Wilcoxon signed-rank analysis for paired primary and metastatic tissues.

Group	Marker	n	Z	*p*
Overall paired cohort	VASH1	45	−3.094	0.002
	VEGF	45	−3.652	<0.001
	CD34-defined MVD	45	−4.476	<0.001
Adjuvant recurrence	VASH1	28	−2.542	0.011
	VEGF	28	−2.832	0.005
	CD34-defined MVD	28	−4.126	<0.001
De novo metastatic	VASH1	17	−1.782	0.075
	VEGF	17	−2.382	0.017
	CD34-defined MVD	17	−1.874	0.061

Abbreviations: CD34: cluster of differentiation 34; MVD: microvessel density; VASH1: vasohibin-1; VEGF: vascular endothelial growth factor.

**Table 5:** Univariate and multivariate Cox analyses investigating the associations between potential prognostic variables and OS.

	Univariate		Multivariate	
Variable	HR (95% CI)	*p* Value	HR (95% CI)	*p* Value
Age, years (≥65 vs. <65)	0.73 (0.42–1.27)	0.274	NA	NA
Gender (male vs. female)	0.96 (0.60–1.51)	0.860	NA	NA
Tumor location (right vs. left)	0.80 (0.45–1.40)	0.441	NA	NA
Distant metastasis (present vs. absent)	3.51 (2.18–5.64)	<0.001	2.92 (1.75−4.88)	<0.001
*KRAS* mutation status (mutant vs. wild)	1.16 (0.73–1.83)	0.517	NA	NA
LVI (present vs. absent)	1.42 (0.85–2.37)	0.173	1.21 (0.71−2.06)	0.471
PNI (present vs. absent)	1.15 (0.70–1.89)	0.571	NA	NA
Histology (moderate-poor vs. good)	1.71 (1.07–2.71)	0.023	1.16 (0.71−1.91)	0.541
VASH1 expression (high vs. low)	4.37 (2.61–7.30)	<0.001	2.05 (1.01−4.20)	0.048
CD34-defined MVD (high vs. low)	3.59 (2.16–5.95)	<0.001	2.12 (1.15−3.89)	0.015
VEGF expression (high vs. low)	1.85 (1.16–2.95)	0.009	1.16 (0.66−2.03)	0.592

Abbreviations: CD34: cluster of differentiation 34; CI: confidence interval; HR: hazard ratio; LVI: lymphovascular invasion; MVD: microvessel density; NA: not applicable; OS: overall survival; PNI: perineural invasion; VEGF: vascular endothelial growth factor; VASH1: vasohibin-1.

**Table 6:** Sensitivity multivariable Cox regression analysis for OS using median-based cut-offs.

Variable	HR (95% CI)	*p* Value
Distant metastasis (present vs. absent)	2.86 (1.71–4.77)	<0.001
LVI (present vs. absent)	1.23 (0.72–2.09)	0.448
Histology (moderate-poor vs. good)	1.18 (0.72–1.93)	0.512
VASH1 expression (high vs. low, median-based)	2.11 (1.04–4.31)	0.039
CD34-defined MVD (high vs. low, median-based)	2.03 (1.12–3.68)	0.020
VEGF expression (high vs. low, median-based)	1.17 (0.67–2.04)	0.592

Abbreviations: CD34: cluster of differentiation 34; CI: confidence interval; HR: hazard ratio; LVI: lymphovascular invasion; MVD: microvessel density; OS: overall survival; VEGF: vascular endothelial growth factor; VASH1: vasohibin-1.

## Data Availability

The datasets generated and/or analyzed during the current study are available from the corresponding author on reasonable request, subject to patient privacy and ethical considerations.

## Abbreviations

The following abbreviations are used in this manuscript:

AJCC	American Joint Committee on Cancer
AUC	area under the curve
CD31	cluster of differentiation 31
CD34	cluster of differentiation 34
CI	confidence interval
CMS	consensus molecular subtype
CRC	colorectal cancer
EGFR	epidermal growth factor receptor
FFPE	formalin-fixed paraffin-embedded
H&E	hematoxylin–eosin
HR	hazard ratio
IHC	immunohistochemistry
IQR	interquartile range
LVI	lymphovascular invasion
MSS	microsatellite-stable
MVD	microvessel density
NA	not applicable
OS	overall survival
PI3K	phosphoinositide 3-kinase
PNI	perineural invasion
ROC	receiver operating characteristic
SPSS	Statistical Package for the Social Sciences
TGF-β	transforming growth factor beta
TMA	tissue microarray
TNM	Tumor–Node–Metastasis
VASH1	vasohibin-1
VEGF	vascular endothelial growth factor
VEGF-A	vascular endothelial growth factor A
